# A comparison of lateral release rates in fixed- versus mobile-bearing total knee arthroplasty

**DOI:** 10.1007/s10195-015-0338-y

**Published:** 2015-02-17

**Authors:** K. B. Ferguson, O. Bailey, I. Anthony, P. J. James, I. G. Stother, M. J. G. Blyth

**Affiliations:** 1 Department of Orthopaedics and Trauma, Glasgow Royal Infirmary, 84 Castle Street, Glasgow, G4 0SF Scotland UK; 2 Department of Orthopaedics and Trauma, Nottingham City Hospital, Nottingham, UK

**Keywords:** Knee arthroplasty, Lateral release, Mobile bearing

## Abstract

**Background:**

With increasing functional demands of patients undergoing total knee arthroplasty, mobile-bearing (MB) implants were developed in an attempt to increase the functional outcome of such patients. In theory, with MB implants, the self-alignment should reduce the rate of lateral release of the patella, which is usually performed to optimise patellofemoral mechanics. This study reports on the lateral release rates for the P.F.C. Sigma® MB posterior-stabilised total knee replacement (TKR) implant compared with its fixed-bearing (FB) equivalent.

**Materials and methods:**

A total of 352 patients undergoing TKR were randomly allocated to receive either MB (176 knees) or FB (176 knees) posterior-stabilised TKR. Further sub-randomisation into patellar resurfacing or retention was performed for both designs. The need for lateral patellar release was assessed during surgery using a ‘no thumb technique’, and after releasing the tourniquet if indicated.

**Results:**

The lateral release rate was the same for FB (10 %) and MB implants (10 %) (*p* = 0.9). However, patellar resurfacing resulted in lower lateral release rates when compared to patellar retention (6 vs 14 %; *p* = 0.0179) especially in MB implants (3 %).

**Conclusions:**

It has been previously reported that alterations to the design of the P.F.C. system with a more anatomical trochlea in the femoral component improved patellar tracking. The addition of a rotating platform tibial component to the P.F.C. Sigma system has, on its own, had no impact on the lateral release rate in this study. Optimising patellar geometry by patellar resurfacing appears more important than tibial-bearing design. Although MB implants appear to reduce the need for lateral release in the P.F.C. Sigma Rotating Platform, this only occurs when the patellar geometry has been optimised with patellar resurfacing.

**Level of evidence:**

Level 2.

## Introduction

Fixed-bearing (FB) total knee arthroplasty is a successful operation with well-documented excellent long-term results [1, 2]. However, because of changing demographics in patients who require total knee arthroplasty, i.e., shifting to a younger population with higher functional demands, newer designs have been developed to achieve greater survivorship and clinical outcomes.

Mobile bearings (MBs) were designed to reduce the peak loading stress and backside wear observed as a cause of aseptic loosening in FB designs [3]. To achieve this, they have a more conforming superior articular surface which, in theory, reduces the contact stresses [1, 4–8]. The introduction of a second bearing interface results in a decoupling of the complex multidirectional motions which occur in FB designs producing unidirectional motion at the two bearing interfaces of the MB implant which, in theory, should reduce polyethylene wear. There have been concerns raised, however, about the risk of MB dislocation and some reports of early backside wear in some clinical studies [1, 7].

In addition, MB designs have the potential to correct any rotational malalignment of the femoral and tibial components by allowing the patellar tendon to self-align throughout a range of motion, enhancing both patellofemoral and tibiofemoral mechanics [9]. Little attention has been given to the potential effects that this decoupling may have on the patellofemoral joint portion of the articulation. In theory, the self-alignment seen with MB designs should reduce the rate of lateral release of the patella, which is usually performed to optimise patellofemoral mechanics.This study reports on the lateral release rates for P.F.C.^®^ Sigma MB posterior-stabilised TKR compared with its FB equivalent.

## Materials and methods

Three hundred and fifty-two patients were randomised to receive a PFC Sigma© total knee replacement (TKR) with either FB or MB implants. The randomisation occurred at the pre-operative assessment stage with the inclusion of patients who had a pre-operative diagnosis of osteoarthritis. Patients who had undergone previous knee surgery, inflammatory arthropathy or had a significant co-morbidities were excluded from the trial.

The study was granted full ethical approval from the Multisite Research Ethics Committee and the Local Research Ethics Committee. Informed consent was obtained from each patient following a full explanation and provision of all necessary patient information.

A single knee design was used in this study (PFC Sigma® Posterior Stabilised; DePuy Inc., Warsaw, IN, USA) with all components being cemented using Palacos®cement. The femoral component was constant for all patients with the tibial component being randomised into two main groups (MB vs FB) using a third party computerised randomisation process.

**Fig. 1:**
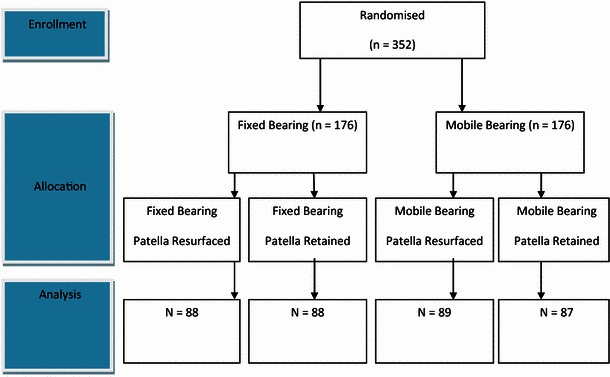
CONSORT diagram

Each patient was randomised into receiving either FB or MB prosthesis, and sub-randomisation was performed to determine whether the patella would be resurfaced or not. The need for lateral release was determined at the time of surgery using a ‘no thumb technique’ and releasing the tourniquet if required. Lateral release was performed where tilting or subluxation of the patella occurred as the knee was taken through a range of motion, before retinacular closure. There was no difference between lateral release rates between surgeons (Fig. 1).

The surgical details of the 352 patients recruited into the trial were used. The two groups were matched for age, sex and body mass index (Table 1). Statistical analysis of the data was performed by an independent statistician. For normally distributed data, the two-sample *t*-test was used. Where the data had unequal variance or was not normally distributed, the Wilcoxon rank sum test was used.

**Table 1:** Cohort demographics

	Fixed-bearing implants	Mobile–bearing implants	*p* value
Patients (*n*)	176	176	
Age			
Mean (years)	69.8 (8.16)	70.2 (7.60)	0.70*
(SD)			
Range	42–89	52–89	
Gender			
Female (*n*) (%)	94 (53 %)	93 (53 %)	1.0^+^
Male (*n*) (%)	82 (47 %)	83 (47 %)	
ASA			
I (*n*) (%)	66 (38 %)	47 (27 %)	0.03^!^
II (*n*) (%)	100 (57 %)	111 (63 %)	
III (*n*) (%)	9 (5 %)	18 (10 %)	
No data (*n*)	1 (1 %)	0	
Body mass index			
Mean (kg/m^2^) (SD)	29.7 (4.9)	31.1 (5.0)	0.28*

* *p* value based on a two-sample *t*-test with unequal variance

^!^*p* value based on chi-squared test

^+^*p* value based on Fisher’s exact test

## Results

The lateral release rate was the same for both the FB and MB designs with 17 patients in each group requiring lateral release (10 %) (*p* = 0.9) (Table 2).

**Table 2:** Lateral release rates of fixed versus mobile bearings

	No release *n* (%)	Lateral release *n* (%)	Medial release *n* (%)	Other *n* (%)
Fixed (*n* = 176)	159 (90)	17 (10)	0 (0)	0 (0)
Mobile (*n* = 176)	158 (90)	17 (10)	0 (0)	1 (<1)*
*p* value		*p* = 0.9		

* Posterior release

There was a statistically significant difference, however, in the lateral release rates between the patients who had their patella resurfaced and those who did not (6 vs 14 %) (*p* = 0.0179) (Table 3).

**Table 3:** Lateral release rates of patella resurfacing versus retention

	No release *n* (%)	Lateral release *n* (%)	Medial release *n* (%)	Other *n* (%)
Patella resurfaced (*n* = 176)	166 (94)	10 (6)	0 (0)	0 (0)
Patella retained (*n* = 176)	151 (86)	24 (14)	0 (0)	1 (<1)*
*p* value		*p* = 0.0179		

Closer analysis of the data including sub-randomisation of the bearing type, revealed an insignificant difference in the lateral release rates between those who had patellar resurfacing and those who did not in the FB group (8 vs 11 %) (*p* = 0.4). However, there was a significantly lower rate of lateral release in the patients in the MB group who had patellar resurfacing compared to those who did not (3 vs 16 %) (*p* = 0.009) (Table 4).

**Table 4:** Lateral release rates of fixed versus mobile versus patella

Bearing	Patella	No release *n* (%)	Lateral release *n* (%)	Medial release *n* (%)	Other *n* (%)
Fixed	Resurfaced (*n* = 89)	81 (92)	7 (8)	0 (0)	0 (0)
	Retained (*n* = 88)	78 (89)	10 (11)	0 (0)	0 (0)
*p* value			*p* = 0.44		
Mobile	Resurfaced (*n* = 88)	85 (96)	3 (3)	0 (0)	0 (0)
	Retained (*n* = 89)	73 (84)	14 (16)	0 (0)	1 (<1)
*p* value			*p* = 0.009		

## Discussion

Lateral release has been performed with FB TKA to optimise patellar tracking [10]; however, it is not without complications by jeopardising soft tissue and wound healing [10, 11]. Lateral release has also been proposed as a cause of avascular necrosis of the patella by interrupting the blood supply [11, 12]. Scuderi et al. demonstrated a higher incidence of vascular compromise to the patella when lateral release was performed [12]. If MB reduces the lateral release rate it may therefore reduce the rate of these complications.

In MB total knee arthroplasty there is potential for self-alignment of the bearing with the femoral component [13]. In an FB design that is inserted with internal rotation of the tibial component, the tibial tubercle becomes lateralized; however, with an MB design the self-alignment potentially permits correction in this circumstance [9, 13]. Rees et al. [14] provided evidence in support of this theory with in vivo fluoroscopic studies. Sawaguchi et al. [13] demonstrated in an intra-operative kinematic study that there was significantly improved patellar tracking with decreased patellofemoral contact stresses. Despite this theoretical advantage, there is no evidence as yet to demonstrate better clinical outcomes [9].

Design improvements of the femoral component of the PFC Sigma® system created a more anatomic trochlear groove that has favourably enhanced patella tracking [15]. In this study, Ballantyne et al. demonstrated a lateral release rate of 15.1 % for the newer FB PFC Sigma® design [15] compared to the older press-fit condylar prosthesis in prospective groups of patients. The addition of a rotating platform tibial component had no impact on the lateral release rate in our study; however, there was a statistically significant positive advantage for patellar resurfacing. This suggests that it is patellofemoral congruency rather than patellofemoral alignment that determines the need for lateral release in TKR.

The data also show the positive effects of patellar resurfacing and MB TKR which together gave the lowest lateral release rates of all groups. Perhaps the benefits offered by the rotating platform design which allows self-alignment of the patella are only realised once the patellofemoral geometry has been optimised. We believe that patellar resurfacing may therefore reduce the need for lateral release in MB knees and should be considered when tracking is suboptimal at the time of assessment with trial components in situ.

## References

[1] Bhan S, Malhotra R, Kiran EK, Shukla S, Bijjawara M (2005). A comparison of fixed-bearing and mobile-bearing total knee arthroplasty at a minimum follow-up of 4.5 years. J Bone Joint Surg Am.

[2] Kim YH, Kim JS, Choe JW, Kim HJ (2012). Long-term comparison of fixed-bearing and mobile-bearing total knee replacements in patients younger than fifty-one years of age with osteoarthritis. J Bone Joint Surg Am.

[3] Ball ST, Sanchez HB, Mahoney OM, Schmalzried TP (2011). Fixed versus rotating platform total knee arthroplasty: a prospective, randomized, single-blind study. J Arthroplast.

[4] Argenson JN, Parratte S, Ashour A, Saintmard B, Aubaniac JM (2012). The outcome of rotating-platform total knee arthroplasty with cement at a minimum of ten years of follow-up. J Bone Joint Surg Am.

[5] Jolles BM, Grzesiak A, Eudier A, Dejnabadi H, Voracek C, Pichonnaz C (2012). A randomised controlled clinical trial and gait analysis of fixed- and mobile-bearing total knee replacements with a five-year follow-up. J Bone Joint Surg Br.

[6] Watanabe T, Tomita T, Fujii M, Hashimoto J, Sugamoto K, Yoshikawa H (2005). Comparison between mobile-bearing and fixed-bearing knees in bilateral total knee replacements. Int Orthop.

[7] Bhatt H, Rambani R, White W, Chakrabarty G (2012). Primary total knee arthroplasty using the P.F.C Sigma®-rotating platform cruciate retaining endoprosthesis—a 6 year follow up. Knee.

[8] Stukenborg-Colsman C, Ostermeier S, Hurschler C, Wirth CJ (2002). Tibiofemoral contact stress after total knee arthroplasty: comparison of fixed and mobile-bearing inlay designs. Acta Orthop Scand.

[9] Pagnano MW, Trousdale RT, Stuart MJ, Hanssen AD, Jacofsky DJ (2004). Rotating platform knees did not improve patellar tracking: a prospective, randomized study of 240 primary total knee arthroplasties. Clin Orthop Relat Res.

[10] Laskin RS (2001). Lateral release rates after total knee arthroplasty. Clin Orthop Relat Res.

[11] Lewonowski K, Dorr LD, McPherson EJ, Huber G, Wan Z (1997). Medialization of the patella in total knee arthroplasty. J Arthroplast.

[12] Scuderi G, Scharf SC, Meltzer LP, Scott WN (1987). The relationship of lateral releases to patella viability in total knee arthroplasty. J Arthroplast.

[13] Sawaguchi N, Majima T, Ishigaki T, Mori N, Terashima T, Minami A (2010). Mobile-Bearing total knee arthroplasty improves patellar tracking and patellofemoral contact stress. In vivo measurements in the same patients. J Arthroplast.

[14] Rees JL, Beard DJ, Price AJ, Gill HS, McLardy-Smith P, Dodd CA (2005). Real in vivo kinematic differences between mobile-bearing and fixed-bearing total knee arthroplasties. Clin Orthop Relat Res.

[15] Ballantyne A, McKinley J, Brenkel I (2003). Comparison of the lateral release rates in the press fit condylar prosthesis and the PFC Sigma prosthesis. Knee.

